# Lycopene Effects on Serum Mineral Elements and Bone Strength in Rats

**DOI:** 10.3390/molecules17067093

**Published:** 2012-06-11

**Authors:** Haidong Liang, Fang Yu, Zhihong Tong, Weifeng Zeng

**Affiliations:** 1 Hands and Feet Microsurgery, Dalian Municipal Central Hospital, Dalian 116033, China; 2 School of Medicine, Dalian University, Dalian 116033, China

**Keywords:** lycopene, biomarkers, BGP, maximum load, maximum stress

## Abstract

This study investigated the beneficial effect of lycopene on bone biomarkers in ovariectomized (OVX) rats. Female Wistar rats were either sham operated or surgically ovariectomized and then fed with lycopene for 8 weeks. Serum Ca, P, alkaline phosphatase (ALP), interleukin 6 (IL-6) and bone gla protein (BGP) concentration was significantly higher in the untreated OVX group compared with that of the sham group, whereas serum estrogen levels were lower. Bone mineral density (BMD), BMD/wt, bone mineral content (BMC), BMC/wt values, maximum load, stiffness, energy and maximum stress were significantly lower in the untreated OVX group compared with that of the sham group. Administration of lycopene (20, 30 and 40 mg/kg b.w.) for 8 weeks significantly decreased serum Ca, P, ALP, and IL-6 concentration, and enhanced serum estrogen level, BMD, BMD/wt, BMC, BMC/wt values, maximum load, stiffness, energy and maximum stress in lycopene-treated OVX groups. In conclusion, the consumption of lycopene may have the most protective effect on bone in OVX rats.

## 1. Introduction

Osteoporosis (a disease of aging associated with bone loss that often occurs without symptoms until microarchitectural deterioration becomes so significant that bone fracture occurs invariably at the trabeculae where a reduction in the bone mass is below what is required for normal bone support), is of great importance in health care. “Although reduced in mass, the bones are normal with respect to mineralization but histologically, there is a decrease in the thickness of the cortex and the number and size of tubeculae of the coarse cancellous bone” [1,2,3]. Plenty of studies around this disease have been carried out in recent years. Accumulating data have indicated that the loss of estrogen at menopause is a major contributor to pathogenesis of the disease because this hormone is a principal negative regulator of osteoclast activity [4,5,6], and osteoclasts are the chief effector’s cells responsible for bone remodeling in osteoporosis [7].

Lycopene is an open-chain, unsaturated carotenoid that imparts red color to tomatoes and other fruits. It is found in unprocessed foods predominantly as the *trans* isomer (CAS No. 502-65-8). As a natural antioxidant, lycopene is associated with the prevention of some chronic diseases including cardiovascular diseases, prostate, and gastrointestinal tract cancers in the human [8,9]. In addition to antioxidant properties, lycopene also possesses some other biologic activities [10,11,12,13] such as control of tumor cell proliferation, reduction of cellular DNA injuries, and regulation of gap junction communications between cells. Moreover, lycopene has been reported for some benefical actions in bone diseases [14,15,16]. Sahni *et al.*’s works suggest a protective role of several carotenoids for bone health in older adults [17] and a protective associations against 4-y loss in trochanter BMD in men and in lumbar spine in women [18]. Rao *et al.* report that the dietary antioxidant lycopene reduces oxidative stress and the levels of bone turnover markers in postmenopausal women [19]. In addition, Mackinnon *et al*.’s work show that the daily consumption of lycopene decrease oxidative injury, bone resorption and increase intake of calcium, niacin, and vitamins A, D, and K in postmenopausal women [20,21,22,23]. Therefore, lycopene may moderate the risk of osteoporosis.

Ovariectomized rats and dogs have been used extensively in osteoporosis models. In 1996, the effects of incadronate on ovariectomized dogs [24] were reported. The purpose of this paper is to establish in the ovariectomized mature rat model of postmenopausal osteoporosis and investigate the effect of lycopene on bone loss and bone strength as measured by mechanical testing.

## 2. Results

There was no statistical difference in initial body weight between groups. Compared with the sham control, final body weight in untreated model group was significantly (*p* < 0.01) enhanced. Final body weights in all doses of lycopene-treated (OVX + lycopene (20, 30 and 40 mg/kg b.w.)) groups were significantly lower than those in the untreated model group (*p* < 0.05; *p* < 0.01) (Table 1).

**Table 1:** Effect of lycopene on initial and final body weight in experimental rats.

Group	Initial weight (g)	Final weight (g)
Sham	228.3 ± 21.5	284.2 ± 24.8
Untreated model (OVX)	227.1 ± 20.6	320.1 ± 26.8 **
OVX + lycopene (20 mg/kg b.w.)	222.3 ± 25.4	279.7 ± 30.1
OVX + lycopene (30 mg/kg b.w.)	223.9 ± 27.1	280.1 ± 32.4 ^#^
OVX + lycopene (40 mg/kg b.w.)	227.4 ± 22.9	282.3 ± 28.4 ^##^

** *p* < 0.01, compared with sham group; ^#^ *p* < 0.05; ^##^ *p* < 0.01, compared with untreated model group.

The serum Ca, P and ALP were higher in untreated model control *vs*. sham control (*p* < 0.01). The Lycopene supplemented [OVX + lycopene (20, 30 and 40 mg/kg b.w.)] groups had lower Ca, P and ALP *vs*. untreated OVX group (*p* < 0.05; *p* < 0.01) (Table 2). In this study, increased ALP activity in OVX group suggested that the animal model is high turnover osteoporosis model. The lower ALP value in lycopene-supplemented groups suggested that lycopene may resist high bone turnover of OVX rats, improve metabolism balance of bone and consequently inhibit high turnover osteoporosis development.

**Table 2:** Effect of lycopene on serum Ca, P and ALP in experimental rats.

Group	Ca (mmol/L)	P (mmol/L)	ALP (U/L)
Sham	2.4 ± 0.17	1.39 ± 0.2	60.53 ± 4.84
Untreated model (OVX)	2.61 ± 0.19 **	1.61 ± 0.18 **	98.37 ± 6.92 **
OVX + lycopene (20 mg/kg b.w.)	2.55 ± 0.22 ^#^	1.56 ± 0.18	83.4 ± 7.32 ^#^
OVX + lycopene (30 mg/kg b.w.)	2.52 ± 0.21 ^#^	1.48 ± 0.16 ^#^	79.22 ± 6.83 ^#^
OVX + lycopene (40 mg/kg b.w.)	2.45 ± 0.25 ^##^	1.42 ± 0.19 ^##^	71.33 ± 8.28 ^##^

** *p* < 0.01, compared with sham group; ^#^ *p* < 0.05; ^##^ *p* < 0.01, compared with untreated model group.

Compared with sham control, serum estrogen level was markedly (*p* < 0.01) decreased, whereas IL-6, BGP and CTx levels were significantly increased in untreated model group. Lycopene treatment (20, 30 and 40 mg/kg b.w.) significantly (*p* < 0.05; *p* < 0.01) dose-dependently enhanced serum estrogen level and decreased serum IL-6, CTx levels in OVX + lycopene (20, 30 and 40 mg/kg b.w.) groups compared to untreated OVX group (Table 3). BGP can sustain normal bone calcification rate and inhibit abnormal hydroxyapatite crystallization formation. In this study, although there wasn’t significant difference in serum BGP level between OVX + lycopene (20 and 30 mg/kg b.w.) groups, a dose-dependent effect can still be observed. In high dose (40 mg/kg b.w.), lycopene had a marked effect on BGP.

**Table 3:** Effect of lycopene on serum estrogen, IL-6 and BGP levels in experimental rats.

Group	Estrogen (pmol/L)	IL-6 (pg/mL)	BGP (ng/mL)	CTx (ng/mL)
Sham	80.11 ± 7.95	15.73 ± 1.66	23.16 ± 2.68	60.38 ± 5.71
Untreated model (OVX)	56.29 ± 6.32 **	74.08 ± 8.29 **	36.11 ± 4.57 **	98.04 ± 7.62 **
OVX + lycopene (20 mg/kg b.w.)	65.28 ± 7.04	61.44 ± 7.43 ^#^	37.25 ± 4.92	88.57 ± 6.92 ^#^
OVX + lycopene (30 mg/kg b.w.)	71.42 ± 8.38 ^##^	47.29 ± 5.62 ^##^	34.16 ± 3.72	81.42 ± 8.03 ^##^
OVX + lycopene (40 mg/kg b.w.)	77.28 ± 8.11 ^##^	25.05 ± 3.33 ^##^	29.27 ± 3.48 *	74.33 ± 5.81 ^##^

* *p* < 0.05, ** *p* < 0.01, compared with sham group; ^#^ *p* < 0.05; ^##^ *p* < 0.01, compared with untreated model group.

Compared with sham control, BMD, BMD/wt, BMC and BMC/wt values were markedly (*p* < 0.05; *p* < 0.01) decreased in untreated model group. Lycopene treatment (20, 30 and 40 mg/kg b.w.) significantly (*p* < 0.05; *p* < 0.01) dose-dependently enhanced BMD/wt, and BMC/wt values in OVX + lycopene (20, 30 and 40 mg/kg b.w.) groups compared to untreated OVX group (Table 4). Lycopene treatment (30 and 40 mg/kg b.w.) significantly (*p* < 0.05; *p* < 0.01) dose-dependently enhanced BMD, and BMC values in OVX + lycopene (30 and 40 mg/kg b.w.) groups compared to untreated OVX group (Table 4).

**Table 4:** Effect of lycopene on BMD, BMD/wt, BMC and BMC/wt values in experimental rats.

Group	BMD (g/cm^2^)	BMD/wt (g/cm^2^)/kg	BMC (g)	BMC/wt (g/kg)
Sham	0.162 ± 0.013	0.683 ± 0.059	0.378 ± 0.021	1.52 ± 0.13
Untreated model (OVX)	0.131 ± 0.011 **	0.538 ± 0.062 **	0.321 ± 0.019 *	1.31 ± 0.11 **
OVX + lycopene (20 mg/kg b.w.)	0.142 ± 0.012	0.582 ± 0.051 ^#^	0.333 ± 0.024	1.41 ± 0.13 ^#^
OVX + lycopene (30 mg/kg b.w.)	0.153 ± 0.011 ^#^	0.637 ± 0.066 ^##^	0.368 ± 0.027 ^#^	1.48 ± 0.12 ^#^
OVX + lycopene (40 mg/kg b.w.)	0.159 ± 0.013 ^##^	0.679 ± 0.07 ^##^	0.373 ± 0.031 ^##^	1.5 ± 0.12 ^##^

* *p* < 0.05, ** *p* < 0.01, compared with sham group; ^#^ *p* < 0.05; ^##^ *p* < 0.01, compared with untreated model group.

The biomechanical competence of the femoral diaphysis was tested after four weeks of treatment with lycopene by using an *ex vivo* three-point bending test. OVX resulted in a significantly decrease in maximum load, stiffness, energy and maximum stress in femur compared with sham group (*p* < 0.05). Compared with the OVX group, four weeks of treatments with three doses of lycopene (20, 30 and 40 mg/kg b.w.) significantly dose-dependently increased the maximum stress (*p* < 0.05 or 0.01, Table 5) in OVX + lycopene (20, 30 and 40 mg/kg b.w.) groups. Lycopene (40 mg/kg b.w.) significantly (*p* < 0.01) increased maximum load, and energy in OVX + lycopene (40 mg/kg b.w.) group. Maximum load, and energy in rats fed with lycopene (20, and 30 mg/kg b.w.) were slightly (*p* > 0.05) increased compared with the OVX group. Lycopene (20, 30 and 40 mg/kg b.w.) has no significant effect on stiffness in OVX + lycopene (20, 30 and 40 mg/kg b.w.) groups.

**Table 5:** Effect of lycopene on maximum load, stiffness, energy and maximum stress in experimental rats.

Group	Maximum load (N)	Stiffness (N/mm)	Energy (N × mm)	Maximum stress (MPa)
Sham	112.17 ± 8.39	164.18 ± 19.65	39.16 ± 4.29	192.37 ± 16.07
Untreated model (OVX)	102.64 ± 7.21 *	155.73 ± 21.65	31.86 ± 4.01 *	143.85 ± 11.26 **
OVX + lycopene (20 mg/kg b.w.)	105.63 ± 8.29	158.38 ± 17.09	35.17 ± 4.72	162.07 ± 10.84 ^#^
OVX + lycopene (30 mg/kg b.w.)	108.47 ± 11.32	160.72 ± 18.36	37.08 ± 5.29	178.49 ± 13.49 ^##^
OVX + lycopene (40 mg/kg b.w.)	111.31 ± 8.17 ^#^	163.25 ± 17.75	38.53 ± 5.22 ^#^	187.39 ± 13.95 ^##^

* *p* < 0.05, ** *p* < 0.01, compared with sham group; ^#^ *p* < 0.05; ^##^ *p* < 0.01, compared with untreated model group.

## 3. Discussion

In the present study, we examined the beneficial effects of lycopene against the reduction of bone mass, mineral elements and bone strength induced by ovariectomy in rats.

As seen in many studies, ovariectomized rats have significantly higher body weights compared to sham-operated rats due to fat deposition caused by estrogen deficiency [25,26,27]. In this study, as expected, this excess body weight gain was completely prevented by lycopene administration. We supposed that lycopene modulate hormone metabolism balance, and improve body energy metabolism balance, therefore, prevent abnormal weight gain.

Increasing the intake of fruits and vegetables (by virtue of the contents of polyphenolic compounds, and flavonoid antioxidants) has been suggested to help improve bone health against oxidative damage and osteoporosis [28,29,30,31,32,33,34,35,36]. Chiba *et al*. [37] reported that the citrus flavonoid hesperidin fed to ovariectomized mice prevented ovariectomy induced bone loss. Deyhim *et al*. [28] showed that drinking orange juice or grapefruit juice would improve serum antioxidant status and protect bone against osteoporosis in senescent male rats. Our investigation showed treatment with three doses of lycopene for 8 weeks was able to prevent the increased blood Ca and P induced by the OVX, which indicate that the rate of bone loss is down regulated by lycopene. ALP [38,39] is widely accepted phenotype markers for bone formation. The present study shows treatment with lycopene after 8 weeks on OVX rats induces significant decrease in serum ALP.

IL-6 was expressed in bone marrow stromal cells and osteoblasts. It could promote the formation and differentiation of osteoclast precursors. Estrogen may inhibit IL-6 expression and bone resorption stimulated by IL-6. The bone Gla protein (BGP or osteocalcin) is a vitamin K-dependent calcium-binding protein synthesized by osteoblasts and found primarily in bone (1–2% of total bone proteins). Serum BGP measurement provides a noninvasive specific marker for bone metabolism. In the present study, lycopene treatment could significantly increase serum estrogen and decrease IL-6 levels in OVX rats.

Decreased bone mass is one of the major factors jeopardizing bone integrity, resulting in reduced bone strength and an increased susceptibility to fractures [40]. BMD is the gold standard for the evaluation of individuals at risk for osteoporosis, as it best predicts the fracture risk in people without previous fractures [41]. BMD measurement is widely used for detecting osteoporosis. In the current study, the results manifested that OVX significantly decreased the BMD and BMC of the total femur when compared with the sham group. Lycopene administration prevented OVX-induced bone loss of the total femur.

Biochemical markers of bone turnover have been widely used as research tools to measure the effects of various drugs on bone remodeling [42,43,44]. In the present study, Lycopene administration prevented OVX-induced increased serum CTx level in OVX animals. This indicated that Lycopene possessed the anti-resorptive function of bone. Most often, a three-point bending test of the bone specimens measures the bone strength. Biomechanical tests evaluate maximum load, stiffness, and energy absorption capacity are evaluated, and all of these parameters affect bone fragility [45]. In addition, at the end of eight weeks, the OVX showed a significant reduction in the threepoint bending test of the midshaft of the femur, including maximal load, energy to ultimate load, and linear stiffness. Lycopene-treated groups exhibited superior mechanical bone properties compared to the OVX groups. Biomechanical evaluation indicated that lycopene could improve bone strength in OVX rats.

## 4. Experimental

### 4.1. Material

Lycopene was purchased from Guangzhou Dijie Biology Science Technology Ltd. (Guangzhou, China). Purity was 95%.

### 4.2. Animals and Experimental Procedures

Fifty 2-month-old Wistar female rats (body weight 225.0 ± 10.4 g) were obtained from Experimental Animal Center. (Dalian, China) and were housed in cages that were maintained at 22 °C with a 12-h light/dark cycle. During the experimental period, the rats were maintained on standard rodent chow (Animal Center of Dalian Medical University, Dalian, China) that contained 0.9% calcium and 0.7% phosphate, and distilled water available *ad libitum*. The acclimatized rats were either sham-operated (Sham, n = 10) or bilaterally ovariectomized (OVX, n = 40) using the dorsal approach [46,47]. In brief, the rats were anesthetized with ether. A single longitudinal skin incision was made on the dorsal midline at the level of the kidneys. Both ovaries were ligated and removed. The rats of the sham group underwent sham surgery, during which time the ovaries were exposed and replaced intact. OVX rats were randomly divided into four groups: model (OVX) control group (OVX, n = 10); three lycopene-treated groups (n = 10). The rats of lycopene-treated groups were treated with lycopene 20, 30 and 40 mg/(kg body weight day) dissolved in corn oil respectively by intragastric administration for 8 weeks. Rats in the sham and OVX control groups were administrated with the same volume of corn oil. The treatment started four weeks after the surgery for 12 weeks. Rat body weight was recorded every week to assess changes. Fasting 24-h urine samples were collected by placing animals in individual metabolic cages during the last week.

After sacrifice, blood samples were taken from the abdominal aorta. Then serum samples were obtained by centrifuged at 2,000 × g for 20 min at 4 °C and stored at −70 °C before assessment of biochemical parameters. The success of ovariectomy was confirmed at necropsy by failure to detect ovarian tissue and by observation of marked atrophy of the uterine horns. At the time of death, the femurs were dissected and the right femur was placed in 10% phosphate-buffered formalin for 24 h and transferred to 70% ethanol for next measurements. The left femur was wrapped in gauze saturated with physiological saline and stored at −20 °C before biomechanical analysis. All animals were treated according to the Guide for Care and Use of Laboratory Animals with the approval of Institutional Ethics Committee of the Dalian Medical University on animal experiment.

### 4.3. Analysis of Serum Ca, P Concentration and Serum ALP, IL-6, Estrogen, BGP

Serum calcium (Ca), inorganic phosphates (Pi) concentration and serum alkaline phosphates (ALP) were measured on an automatic analyzer (Ciba-Corning 550, Washington, DC, USA) using diagnostic reagent kit *in vitro* determination. Serum IL-6 was estimated using an Elisa kits according to product instructions. Concentration of blood estrogen was determined by double-antibody RIA kits purchased from the Nanjing JianCheng Bioengineering Ltd. (Najing, China). BGP was measured by an immunoradiometric method.

### 4.4. Determination of Bone Density

Measurements of bone mineral concentration (BMC), bone width (BW) and bone mineral density (BMD = BMC/BW) were made at the middle and epiphysis of femur by dual energy X-ray absorptiometry PIXImus (GE Lunar Co, Madison, WI, USA) at beginning of experiment and 20 weeks after operation.

### 4.5. Three-Point Bending Test

Prior to mechanical testing, the left femurs were slowly thawed and held at room temperature on the day of test, the length of the femurs (distance from intermalleolar to intercondylar region) were measured with a micrometer and the middle of the diaphysis was determined. The intact femur then was placed in the material testing machine on two supports separated by a distance of 20 mm and load was applied to the middle of the diaphysis, thus creating a three-point bending test. The biomechanical quality of the left femoral diaphysis were determined using 858 Mini Bionix II material testing machine (MTS, Eden Prairie, MN, USA) at a speed of 2 mm/min. The central loading point was displaced, and the load and displacement were recorded until the specimen was broken. From the load-deformation curve, maximum load (ultimate strength, Fmax), stiffness (slope of the linear part of the curve representing elastic deformation), and energy absorption (area under the curve, Wabs), maximum stress (Fmax/cross-sectional area, σmax) and Young’s modulus (maximum slope of the stress-strain curve, E) were obtained.

### 4.6. Statistical Analysis

Statistical studies were performed using SAS/Stat Version 6.04 software (SAS Insitute, Cary, NC USA). Data were analyzed by one-way analysis of variance followed by Duncan’s multiple range test. A probability level of 0.05 was selected as the point at which differences were considered significant. Data are presented as means ± SD.

## 5. Conclusions

In the present study, serum Ca, P and ALP, IL-6 and BGP levels were significantly increased, whereas serum estrogen, BMD, BMD/wt, BMC and BMC/wt values, maximum load, stiffness, energy and maximum stress were markedly decreased in ovariectomized rats. Lycopene treatment can inhibit bone loss and increase bone strength in OVX rats.
